# Cervical congenital infantile fibrosarcoma: a case report

**DOI:** 10.1186/s13256-019-1968-0

**Published:** 2019-02-24

**Authors:** Alisha Gupta, Shilpa Sharma, Sandeep Mathur, D. K. Yadav, D. K. Gupta

**Affiliations:** 1 Department of Pediatric Surgery, All India Institute of Medical Sciences, New Delhi, India; 2 Department of Pathology, All India Institute of Medical Sciences, New Delhi, India

**Keywords:** Infantile fibrosarcoma, Neck, Infant

## Abstract

**Background:**

Congenital infantile fibrosarcoma is a rare mesenchymal tumor seen in children as well as adults. The congenital variety is rare and out of the reported cases only one case sited in the neck has been reported so far. Another such case is presented here who was successfully managed.

**Case presentation:**

A 3-month-old Hindu baby boy presented with a congenital neck swelling. The apparent clinical diagnosis was lympho-venous malformation. With a remote possibility of malignancy, an excisional biopsy was done. Histopathology revealed congenital infantile fibrosarcoma.

**Conclusion:**

A successful excision of cervical congenital infantile fibrosarcoma has not been reported. This diagnosis should be kept as a possibility in all congenital cervical swellings. These are commonly misdiagnosed as lympho-venous malformations and histopathology is confirmatory.

## Background

Congenital infantile fibrosarcoma (CIFS) is a rare mesenchymal tumor which, contrary to its name, is seen both in children as well as adults. The congenital variety is even rarer with only a few reported cases. Among the sites of origin of these congenital lesions, occurrence in the neck has been reported only once before [1]. The case reported earlier had died during surgery due to exsanguinating hemorrhage [1]. We report here a successful excision of the lesion. The case is discussed with a review of recent literature.

## Case presentation

**Fig. 1:**
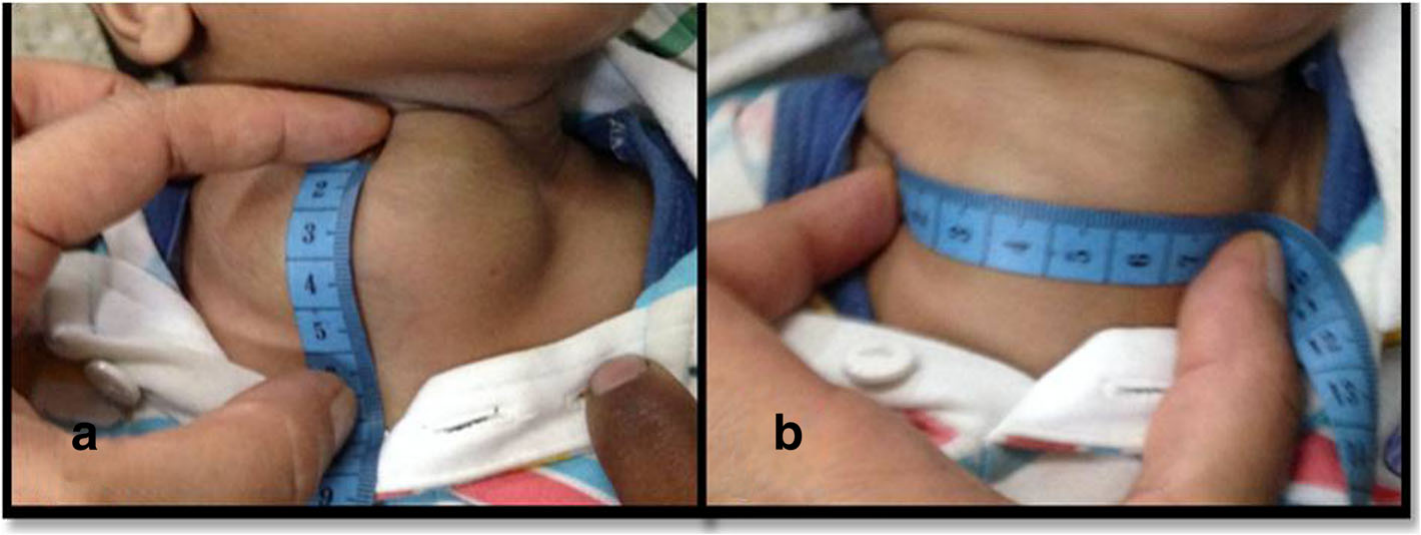
**a** and **b** Clinical photograph showing a 5.5 × 7.5 cm neck mass (right supraclavicular) with bluish hue at places – clinically suspected to be a slow flow vascular malformation

**Fig. 2:**
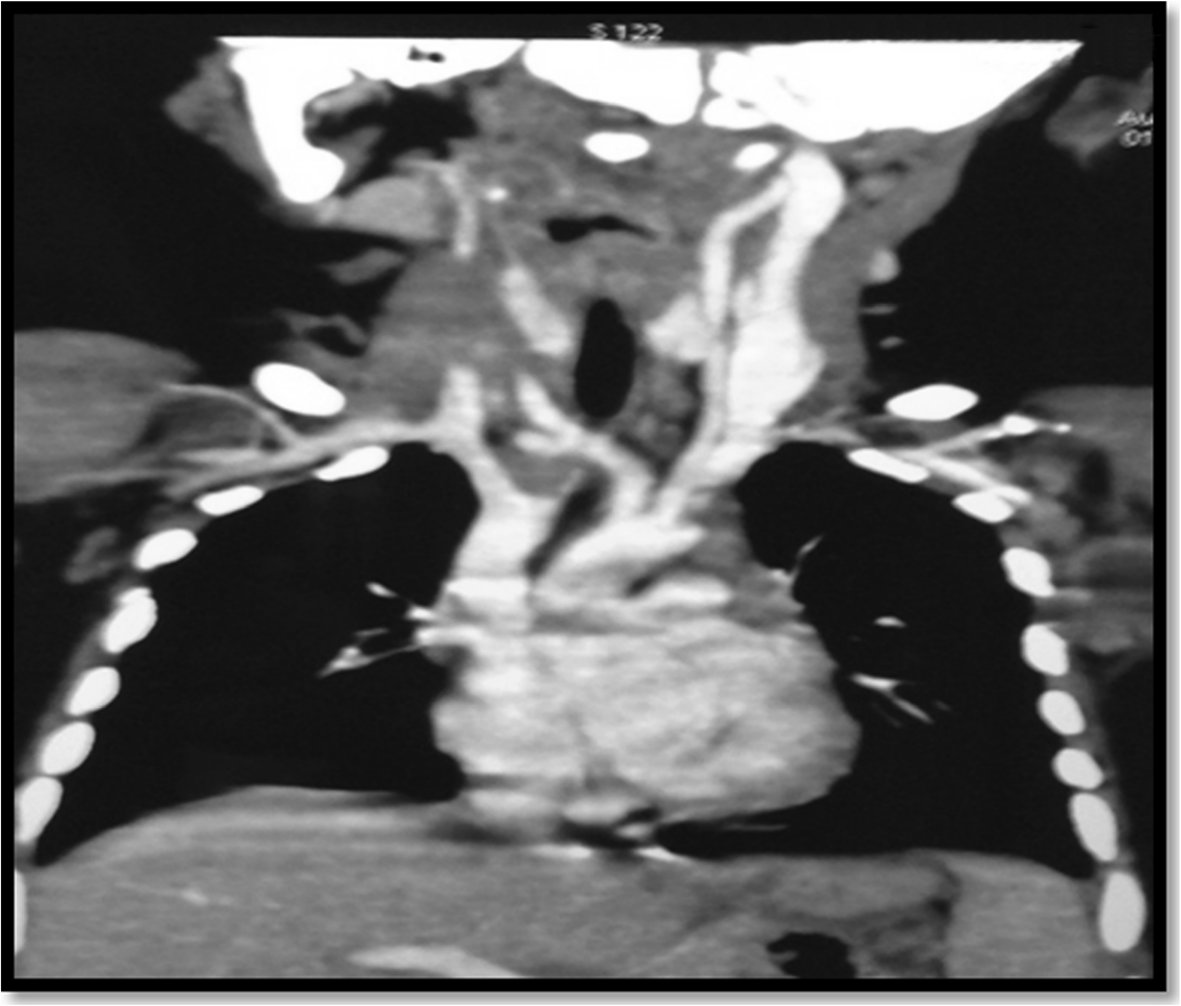
Contrast-enhanced computed tomography of the neck and chest showing a highly vascular heterogenous soft tissue lesion, pushing the neck vessels posteriorly with no retrosternal extension

**Fig. 3:**
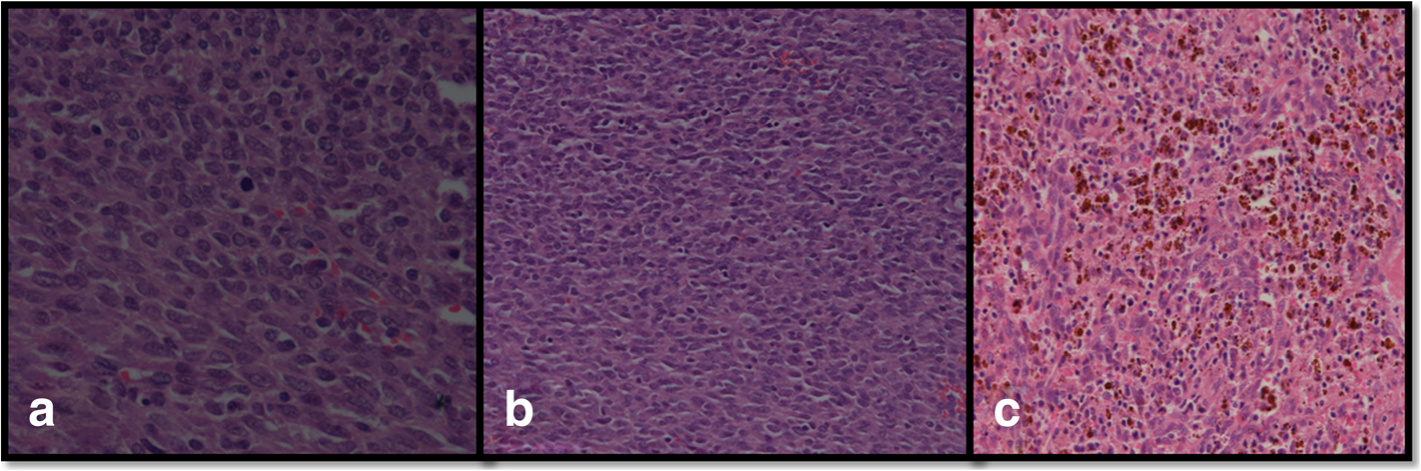
Photomicrographs showing histopathological findings of **a** presence of mitotic figures, **b** spindle-shaped cells, and **c** hemosiderin-laden macrophages

A 3-month-old Hindu baby boy presented with a congenital neck swelling on the right side of his neck. There was no history of birth trauma or breech delivery. Initially a small midline swelling, it progressively increased in size with age. It was soft and compressible with an overlying bluish hue at places. With a working diagnosis of a low flow lymphovascular malformation at another hospital, intralesional bleomycin was injected once after which the swelling became a little firm without any change in its size. One month after the bleomycin injection, it was a 5.5 × 7.5 cm firm, non-tender, well-defined swelling in the midline and extending into the right supraclavicular region (Fig. 1). There was no retrosternal extension and no movement with deglutition or cervical lymphadenopathy. Imaging suggested a diagnosis of lympho-venous malformation (Fig. 2). However, there was a remote suspicion of malignancy as there were interspersed solid areas. Serum alpha-fetoprotein levels were in the normal range for age. On exploration, a friable, solid mass with a pseudocapsule was encountered without any cystic component. It encased the sternal head of right sternocleidomastoid, part of which had to be sacrificed. A frozen section sent during excision was suggestive of malignancy. Complete gross resection of the lesion was done. There were no obviously enlarged neck nodes. Histopathology revealed a tumor comprising spindle-shaped fibroblast-like cells along with large areas of hemorrhage (Fig. 3). Tumor cells were arranged in fascicles and at places in a herringbone pattern. There was brisk mitotic activity and moderate degree of anisonucleosis. Cells were immunopositive for desmin but negative for myogenin, smooth muscle actin (SMA), pancytokeratin, epithelial membrane antigen (EMA), MIC-2, and CD-34. Sternocleidomastoid muscle was free of tumor. The diagnosis of CIFS was favored over spindle cell rhabdomyosarcoma in view of absence of myogenin positivity. A metastatic workup was negative. No chemoradiotherapy was initiated and the child was kept under close follow-up. A follow-up contrast-enhanced computed tomography scan (CECT) of his neck and chest showed no residue or recurrence at 3 and 6 months. He is thriving well and was disease free at 2-year follow-up.

## Discussion and conclusions

CIFS is one of the common non-rhabdomyosarcoma soft tissue tumors in children with an incidence of around 5 per million cases in the pediatric age group [2]. Although only 100 cases were reported until 2009 in children, with ~ 40% present at birth, the entity has been diagnosed in the antenatal period [1, 3, 4]. Although the most common site of occurrence is in the distal extremities, rare locations such as lung, heart, tongue, chest wall, presacral region, and retroperitoneum have also been reported [5–9]. The congenital and adult types of infantile fibrosarcoma, although histologically similar, differ in their clinical presentation in that the adult type has a higher risk of recurrence, higher incidence of metastasis, and fairs poorly in the overall survival.

The differential diagnoses include a wide range of fibroblastic and myofibroblastic tumors. Pediatric fibroblastic and myofibroblastic tumors on the basis of their biologic behavior have been divided into various entities [10]. These have been outlined in Table 1.

**Table 1:** Types of pediatric fibroblastic and myofibroblastic tumors

Group	Types	Characteristics
Benign	Myositis ossificans Myofibroma Fibromatosis colli	Great toe malformations in fibrodysplasia ossificans fibroma, neonatal torticollis in fibromatosis colli
Intermediate-locally aggressive	Lipofibromatosis Desmoid fibroma	Adipose tissue in lipofibromatosis, plaque-like growth pattern of Gardner fibroma
Intermediate-rarely metastasizing	Inflammatory myofibroblastic tumors Infantile fibrosarcoma Low-grade myofibroblastic sarcoma	Low or intermediate signal intensity on T2-weighted magnetic resonance images and extension along fascial planes is seen in fibroblastic or myofibroblastic lesions, multiple subcutaneous or intramuscular lesions may be seen in infantile myofibromatosis
Malignant	Fibromyxoid sarcoma Adult fibrosarcoma	

Composite infantile myofibromatosis of the scalp with several distinct histopathological features including myofibroma, hemangiopericytoma, and fibrosarcoma have been described in newborns [11]. An infantile rhabdomyofibrosarcoma that lies intermediately between rhabdomyosarcoma and infantile fibrosarcoma in terms of clinical presentation, immunohistochemistry, behavior, morphology, and ultrastructural features has been reported in a 26-month-old girl [12].

CIFSs, being vascular lesions, have been notoriously misdiagnosed as hemangiomas and lympho-venous malformations at birth and earlier, as was seen in the present case [1, 13]. Suspicious cutaneous lesions may be diagnosed with a biopsy. Occasional cases of hypercalcemia of malignancy in a newborn with infantile fibrosarcoma have been described [1, 14]. The expression of parathyroid hormone/parathyroid hormone-related protein (PTH/PTH-rP) receptor messenger ribonucleic acid has been detected in the tumor by reverse transcription-polymerase chain reaction (RT-PCR), suggesting the effect of PTH-rP in the tumor in an autocrine/paracrine manner [1]. It has been suggested that in addition to the systemic effect of PTH-rP manifested as hypercalcemia, the PTH-rP secreted from the neoplasm could be a local factor involved in the growth of the tumor and enhanced vascularity leading it to mimic a vascular lesion-like hemangioma [1].

Wide local excision, without any mutilating surgery, is the mainstay of management [15]. In cases of huge tumor size, where functional or anatomical derangement might jeopardize the quality of life in these children, neoadjuvant chemotherapy with vincristine and actinomycin-D (VA) might prove beneficial [3, 16]. In cases, where a tumor-free margin is achieved, close follow-up without any adjuvant chemotherapy is sufficient. Although the role of adjuvant chemotherapy has not been established, chemotherapy has been used postoperatively in cases with positive surgical margins or with residual tumor [17, 18]. In the present case, complete gross excision with tumor-free margin was achieved and hence the child was kept on close follow-up only.

The European Pediatric Soft Tissue Sarcoma Study Group (Intergroup Rhabdomyosarcoma Study; IRS) has developed conservative treatment recommendations according to initial resectability of the tumor [19]. Initial surgery is suggested only if possible without mutilation. Patients with initial complete (IRS group I/R0) or microscopic incomplete (group II/R1) resection have no further therapy. Patients with initial inoperable tumor (group III/R2) receive first-line VA chemotherapy. Delayed conservative surgery is planned after tumor reduction. Aggressive local therapy (mutilating surgery or external radiotherapy) is discouraged. The VA regimen is recommended as the first-line therapy in order to reduce long-term effects [19].

Although CIFSs are biologically more benign than their counterparts occurring in older patients, they are histologically similar. CIFSs belong to one end of a spectrum of fibrous proliferations occurring in children. Gains of chromosomes 8, 11, 17, and 20 (in various combinations) were observed in 11 of 12 fibrosarcomas occurring in infants under 2 years of age [20]. Extra copies of chromosomes 17 and 20 were observed in a fibrosarcoma occurring in a 5-year-old child but no abnormalities were detected by fluorescence *in situ* hybridization (FISH) in four additional fibrosarcomas occurring in patients aged 6–17 years. One of three cellular fibromatoses was characterized by extra copies of chromosome 8, 11, 17, and 20. Similar findings were not observed in any of the noncellular fibromatoses or in myofibromatoses [20]. The ETS variant gene 6-neurotrophin 3 receptor gene (*ETV6*-*NTRK3*) gene fusion product identified by RT-PCR has been recognized as diagnostic of infantile fibrosarcoma. *ETV6*-*NTRK3* transcript was present in 87.2% of patients where the investigation was performed by the European Pediatric Soft Tissue Sarcoma Study Group [19]. Pavlick *et al.* found that 9 out of 2031 advanced cancers from patients less than 21-years old (0.44%) harbored *NTRK* fusions [21]. Notably, four of these cases were in children less than 2-years old for which infantile fibrosarcoma was considered a diagnosis, and two harbored the canonical *ETV6*-*NTRK3* [21]. *NTRK* fusions occur in a subset of young patients with mesenchymal or sarcoma-like tumors at a low frequency, and are potential good targets for drugs. A case of refractory infantile fibrosarcoma (IFS) with constitutive activation of the tropomyosin-related kinase (TRK) signaling pathway from an *ETV6*-*NTRK3* gene fusion experienced a rapid, radiographic response, thus depicting the potential for LOXO-101 (also known as larotrectinib) to provide benefit for IFS harboring *NTRK* gene fusions [22].

Histopathologic characteristics include a solid, dense proliferation of spindle cells in interlacing bundles; positive for vimentin, and occasionally for desmin, SMA, and cytokeratin [23]. Similar findings were observed in the present case. We could not test for the *ETV6*-*NTRK3* gene fusion due to technical resource constraints.

The incidence of metastatic spread of disease is 5–8% [24]. The organs commonly affected in metastasis are the lungs and lymph nodes. Metastatic disease may be demonstrated on fluorodeoxyglucose positron emission tomography-computed tomography [25]. The risk of recurrence is considerably high, being 17–43% [26]. The prognosis is fair with a reported 5-year overall survival rate as high as 84–93% [27].

To conclude, CIFSs should be kept in the differential diagnoses of soft tissue tumors in infants, even in congenital cases. The clinical picture is similar to lymphovascular malformations which might lead to misdiagnosis of these tumors. The mainstay of treatment is complete excision. However, chemotherapy does have a good response and can be a preferred option if surgery is not possible without major anatomical compromise. Overall survival in these tumors is excellent.

## Abbreviations

CECT: Contrast-enhanced computed tomography
CIFS: Congenital infantile fibrosarcoma
EMA: Epithelial membrane antigen
*ETV6*-*NTRK3*: ETS variant gene 6-neurotrophin 3 receptor gene
FISH: Fluorescence *in situ* hybridization
IFS: Infantile fibrosarcoma
IRS: Intergroup Rhabdomyosarcoma Study
PTH/PTH-rP: Parathyroid hormone/parathyroid hormone-related protein
RT-PCR: Reverse transcription-polymerase chain reaction
High school: Smooth muscle actin
TRK: Tropomyosin-related kinase
VA: Vincristine and actinomycin-D
